# Hunger Bias or Gut Instinct? Responses to Judgments of Harm Depending on Visceral State Versus Intuitive Decision-Making

**DOI:** 10.3389/fpsyg.2020.02261

**Published:** 2020-09-18

**Authors:** Helen Brown, Michael J. Proulx, Danaë Stanton Fraser

**Affiliations:** ^1^Crossmodal Cognition Lab, Department of Psychology, University of Bath, Bath, United Kingdom; ^2^CREATE Lab, University of Bath, Bath, United Kingdom

**Keywords:** interoception, moral judgment, hunger, decision-making, moral dilemmas

## Abstract

Empirical investigation into the emotional and physiological processes that shape moral decision-making is vast and growing. Yet, relatively less attention has been paid to measures of interoception in morality research despite its centrality in both emotional and physiological processes. Hunger and thirst represent two everyday interoceptive states, and hunger, in particular, has been shown to be influential for moral decision-making in numerous studies. It is possible that a tendency to focus on internal sensations interoceptive sensibility (IS), as well as the emotional and physiological states associated with visceral states, could be important in the relationships between hunger, thirst, and moral judgments. This cross-sectional online research (*n* = 154) explored whether IS, hunger, thirst, and emotional state influenced appropriateness and acceptability judgments of harm. The moral dilemma stimuli used allowed the independent calculation of (1) people’s tendency to avoid harmful action at all costs and (2) people’s tendency to maximize outcomes that benefit the greater good. The Cognitive Reflection Task (CRT) was implemented to determine whether an ability to override intuitive responses to counterintuitive problems predicted harm-based moral judgments, as found previously. Hunger bias, independent of IS and emotional state, was influential for non-profitable acceptability judgments of harmful actions. Contrary to dual-process perspectives, a novel finding was that more intuitive responses on the CRT predicted a reduced aversion to harmful actions that was indirectly associated with IS. We suggest that IS may indicate people’s vulnerability to cognitive miserliness on the CRT task and reduced deliberation of moral dilemma stimuli. The framing of moral dilemmatic questions to encourage allocentric (acceptability questions) versus egocentric perspectives (appropriateness questions) could explain the divergence between hunger bias and intuitive decision-making for predicting these judgments, respectively. The findings are discussed in relation to dual-process accounts of harm-based moral judgments and evidence linking visceral experiences to harm aversion and moral decision-making.

## Introduction

Our current homeostatic needs provide a context for decision-making (Gailliot, 2013; Yam et al., 2014; Craig, 2015). Important decisions sometimes with serious consequences, such as prescribing antibiotics (Linder et al., 2014), judicial rulings (Danziger et al., 2011), and voting behavior (Gomez et al., 2007), can be influenced by regularly occurring trivialities, such as the time of day (Danziger et al., 2011; Linder et al., 2014), bad weather (Gomez et al., 2007), carbon-dioxide levels (Satish et al., 2012), and how hungry we are (Gailliot, 2013). The connection between how we feel right now and the decisions we make is no coincidence. Interoception refers to our perception and interpretation of visceral sensations associated with homeostatic regulation inside the body, such as those originating in the cardiovascular, respiratory, and gastrointestinal systems (Garfinkel and Critchley, 2013; Craig, 2015). Brain areas responsible for the perception of visceral states (e.g., feeling hot, cold, full) are also implicated in the integration of this information to initiate drive states (e.g., hunger, thirst, sex drive) that in turn affect how we feel (Craig, 2015). The vagus nerve communicates the majority of information from visceral centers to the brain stem (Hellström and Näslund, 2001) coordinating adaptive fight/flight responses on the one hand and emotional expression and social engagement processes on the other, depending on the physiological state of the body (Porges, 1993). There is considerable crossover in brain areas responsible for interoception, emotion, and social cognition (Adolfi et al., 2017), and empirical advances in the field of embodied cognition continue to illuminate how cognitive products of the mind can be rooted within the body (Häfner, 2013). Furthermore, individual differences in how we perceive internal sensations have been shown to be important in the link between visceral processes and decision-making (Dunn et al., 2010; Häfner, 2013).

Visceral states such as hunger can influence ethical decisions in the laboratory (e.g., Yam et al., 2014) and the real world (e.g., Gailliot, 2013). Hunger is the subjective experience of food deprivation comprising visceral sensations in the stomach area, an emotional desire or wanting to eat, and cognitive states associated with eating, food, and hunger (Stevenson et al., 2015). Thirst is a comparatively understudied but related drive, largely regulated by food intake (Mckiernan et al., 2009), and comprises a desire or wanting to obtain and drink water, often accompanied by sensations such as dryness of mouth (Ramsay and Booth, 2012). Incidental emotional states can influence moral decision-making (Valdesolo and Desteno, 2006); sensitivity to moral norms (Gawronski et al., 2018) and emotional-regulation difficulties predict a bias toward immoral judgments (Zhang et al., 2017a). Differences in blood glucose levels have also shown to predict prosocial intentions (Gailliot et al., 2007). Danziger et al. (2011) found the probability of court judges to provide less favorable rulings was increasingly likely before the provision of a food/rest break compared to afterward. However, other researchers (Weinshall-Margel and Shapard, 2011) have contested this, suggesting that the order of cases seen by judges was partly responsible for this observation.

Laboratory-based research has been more effective at substantiating a link between hunger and moral judgments as hunger can be objectively manipulated. Vicario et al. (2018) found hunger reduced moral disapproval ratings for ethical violations, suggesting hunger bias may reduce the harshness of moral judgments. A dispositional sensitivity toward feelings of disgust was also found to increase the severity of moral disapproval ratings of ethical violations. Vicario and colleagues suggested hormonal reactions and interoceptive signals triggered by eating may evoke feelings of nausea interpreted as disgust (Tracy et al., 2019), which subsequently inform moral judgments. This is consistent with other work (Wheatley and Haidt, 2005; Horberg et al., 2009), including Schnall et al. (2008), who found disgust manipulations encourage harsher judgments of ethical violations and is strongest for those with a greater tendency to pay attention to interoceptive sensations. Despite large variations in interoceptive sensitivities between people and daily fluctuations in interoceptive states, individual differences in interoception is an underexplored area in the link between moral decision-making and visceral states such as hunger (Dunn et al., 2006).

Damasio’s et al. (1996) somatic marker hypothesis (SMH) was among the first theoretical frameworks to reveal the neuropsychological foundations that connect fundamental visceral processes with higher-level moral cognitions. The SMH (Damasio et al., 1996) describes how changes in bodily states have the potential to alter our emotional state and bias our thinking processes to support adaptive behavioral responses to the environment (Craig, 2015; Barrett, 2016). The ventromedial prefrontal cortex is believed to be responsible for the representation of homeostatic information (including emotional state) when evaluating ethical violations (Damasio, 1994; Moretto et al., 2010). Damage to this area is associated with emotional deficits in guilt and empathy (Anderson et al., 2013), reduced physiological responses to moral decisions, and greater acceptance of moral violations (Moretto et al., 2010). The insula is a key center for interoceptive integration (Adolfi et al., 2017) and is implicated in processing negative emotional states, particularly disgust sensitivity (Calder et al., 2007), which can bias moral decision-making (Greene et al., 2004). It is possible people with superior ability to perceive interoceptive processes could be more influenced by this information when forming moral judgments. For example, in research using the Iowa Gambling Task (a card-choosing task measuring decision-making under uncertainty), people with a superior ability to detect internal sensations were more influenced by concurrent somatic signals even when those signals unhelpfully guided them toward high-risk card decks (Dunn et al., 2010).

Historically, research exploring emotional influences in moral decision-making has focused on harm-based moral dilemmas such as the Trolley (Thomson, 1985) and Footbridge (Foot, 2003) problems, as particularly emotive moral conflicts to consider (Greene et al., 2001). In these dilemmas, participants judge whether it is acceptable to cause fatal harm to one person either directly (Footbridge) or indirectly (Trolley), as a necessary means to saving the lives of more (>1) people. Judgments can be influenced by an emotional reaction to the harmful *action* toward the one person intentionally harmed (“deontology”) or to the *outcomes* of the action for the many people who would be harmed otherwise (“utilitarianism”) (Cushman, 2013; Miller et al., 2014). This traditional moral dilemma paradigm places utilitarianism and deontology on opposite ends of a bipolar scale, preventing us from determining whether someone chooses to harm the one person because he/she has a weakened aversion to harming others or because he/she is more motivated to save the lives of more people (Conway and Gawronski, 2013). A more recent process-dissociation approach (Conway and Gawronski, 2013) uses moral dilemma stimuli that allow the measurement of people’s outcome-maximization (utilitarian) and harm-aversion (deontological) motivations independently. This method works by calculating the probability that someone chooses to condone harming others when harm results in a “greater good” overall and when it does not. Although people’s tendencies to avoid harm or maximize outcomes do not necessarily represent people’s abstract views about deontological and utilitarian philosophies (Kahane et al., 2018), these terms are used for clarity.

Deontological moral judgments associated with the rejection of harmful action have been associated with more visceral and intuitive decision-making processes than utilitarian decisions (Greene et al., 2001; Park et al., 2016). Greene’s et al. (2004) dual-process account of morality proposes deontological judgments are driven by automatic and emotional responses associated with activation of emotional centers in the brain, whereas utilitarian judgments are driven by more reflective, cognitive processes and are associated with activation of brain areas implicated in cognitive control (Greene et al., 2004). In support of a dual-process conceptualization, emotional arousal predicts deontological preferences (Szekely and Miu, 2015), and performing or witnessing harmful actions correlates with measures of cardiac arousal (Cushman et al., 2012; Parton and McGinley, 2019). More calculative reasoning styles have been associated with utilitarian response tendencies (Patil et al., 2020), and successful performance on the Cognitive Reflection Task (CRT; Frederick, 2005) is associated with increased utilitarian judgments, potentially due to its association with cognitive deliberation (Baron et al., 2015). The CRT task includes questions that have both correct and “intuitive” answers and can be scored according to correct versus intuitive responses (Erceg and Bubić, 2017). Successful performance on this task requires some reflection to avoid the intuitive lures and determine the correct solutions. As such, this task is believed to provide an indication of a person’s ability to “override” their gut response to counterintuitive problems (Frederick, 2005). Byrd and Conway (2019) suggest that arithmetic-reflection ability (captured by the CRT) is responsible for the association with utilitarian preferences, possibly because it indicates a greater numerical focus (i.e., saving *more* lives) when weighing up moral decisions, whereas Park et al. (2016) suggest strong utilitarian preferences may reflect poorer integration of visceral signals into the decision-making process, leading participants to place more weight on the outcomes of harmful action.

The physiological, emotional, and cognitive processes implicated in moral decision-making are relevant to consider in the context of hunger and thirst, as changes in our psychophysiological states have the potential to bias decision-making processes (Critchley and Garfinkel, 2018). Food deprivation is often associated with increased physiological arousal (e.g., Chan et al., 2007; Ribeiro et al., 2009). Ghrelin (the “hunger” hormone) appears to play a role in regulating our responses to stressors potentially by increasing anxiety (see Korbonits et al., 2004) and relationship with the stress hormone cortisol (Sarker et al., 2013). Although there has been less empirical interest in thirst, available evidence suggests hydration levels do not affect cardiovascular reactivity (Schwabe et al., 2007) but can affect blood reactivity to stress (Rochette and Patterson, 2005). Cardiovascular arousal is of particular interest, as arousal represents a core component of emotional experience (Russell and Barrett, 1999), which can intensify the processing of emotionally salient information (McGaugh, 2015) and can influence moral decision-making (Greene et al., 2001). Heartbeat signals alone can directly influence cognition and facilitate the detection of fearful and threatening stimuli (Garfinkel and Critchley, 2016). In addition, the sound of “quickening” heartbeat feedback has shown to predict moral decision-making (Gu et al., 2013), demonstrating how even a belief that we are physiologically aroused can influence our moral choices. Hunger sensations or sensations associated with hunger-induced physiological arousal may manifest as different psychological states (Barrett et al., 2004; MacCormack and Lindquist, 2016), depending on individual differences in perception (Dunn et al., 2010; Herbert et al., 2012) and interpretation (Domschke et al., 2010) of these interoceptive processes. For example, brain regions associated with the conscious awareness of interoceptive states are also implicated in subjective emotional experience (Zaki et al., 2012), and individuals who are better at detecting heartbeat sensations experience more arousal-focused emotional experiences (Barrett et al., 2004). Furthermore, preliminary evidence suggests hunger could actually provide a context for more accurate perception of visceral sensations due to changes in the autonomic nervous system that alter cardiac activity (Herbert et al., 2012). Therefore, although subjective hunger and thirst states may be influential for moral decision-making due to the physiological experiences typically accompanying them, it is likely that individual differences in interoceptive sensitivities will shape how these visceral states translate into psychological and emotional states.

Interoceptive sensibility (IS) is one construct that could influence the psychological manifestation of visceral states and is a measure of a person’s tendency to focus on internal sensations, independent from their ability to objectively detect internal sensations (Garfinkel and Critchley, 2013). Although some evidence suggests heartbeat detection accuracy corresponds with increased sensitivity to bodily information (Duschek et al., 2015), other research indicates interoceptive accuracy and sensibility are unrelated (Ainley and Tsakiris, 2013; Ferentzi et al., 2018). Individual differences in IS have shown to be important in the link between our visceral experiences and subjective appraisals of these experiences (Häfner, 2013) and could potentially shape the interpretation of visceral sensations present during moral decision-making. Individuals high in body awareness typically direct more attention toward visceral sensations, increasing the likelihood they will observe and misinterpret physiological changes as meaningful, which can influence emotional state (Palomba and Stegagno, 1995) and increase anxiety (Clark et al., 1997; Domschke et al., 2010). Paulus and Stein (2010) suggest that visceral sensations detected by people with high levels of anxiety can be intensified and associated with bad or aversive outcomes and is consistent with the finding that IS can increase risk-averse behavior when bodily information is present (Salvato et al., 2019). Overall, the link between anxiety and moral judgments of harm presents a mixed picture. Anxiety facilitates increased vigilance to threats and has been associated with unethical behavior (Kouchaki and Desai, 2015). There is some evidence to suggest that self-oriented anxiety associated with empathy can increase people’s tendency to reject harm in traditional moral dilemmas (Sarlo et al., 2014). Trait anxiety has shown to specifically predict moral goodness ratings of utilitarian action in the Footbridge dilemma, whereas mild anxiety-inducing manipulations appear to have less of an impact on moral judgments (Zhao et al., 2016). It is plausible that a greater attentional focus on bodily sensations could heighten sensitivity to arousal-based physiological sensations accompanying hunger or thirst, which, if interpreted as meaningful and anxiety-evoking (Paulus and Stein, 2010), could influence moral decision-making (Sarlo et al., 2014; Zhao et al., 2016).

Importantly, prior studies exploring the relationship between hunger and moral judgments have measured judgments of ethical violations, which require people to make allocentric judgments about the acceptability of other people’s morally dubious actions (e.g., Vicario et al., 2018). However, moral dilemmas used to explore people’s aversion to harm typically ask questions that facilitate an egocentric perspective, e.g., “Would **you**, carry out X action… in order to?” (e.g., Thomson, 1985; Foot, 2003; Conway and Gawronski, 2013). Several studies have found discrepancies between whether people judge another person’s actions to be morally acceptable and whether people agree that they would perform “immoral” actions themselves (Tassy et al., 2013; Pletti et al., 2017). An egocentric perspective that is putting ourselves in the shoes of the agent committing an immoral act encourages us to consider the self-relevant consequences of our actions (Sood and Forehand, 2005). Egocentric moral judgments, but not allocentric judgments, have been associated with activation of the amygdala, suggesting these judgments rely on emotional processes that allocentric judgments do not (Berthoz et al., 2006). Therefore, it is possible that imagining ourselves personally performing harmful acts could influence how likely we are to refer to bodily and emotional cues when forming moral judgments. Extending previous work, we explored whether the roles of hunger, interoceptive process, and emotional state were associated with moral appropriateness (egocentric) and moral acceptability (allocentric) judgments of harm in the same way. Furthermore, comparing people’s tendency to judge harmful acts as morally acceptable from an allocentric perspective when harm results in a greater good, and when it does not, has not been previously explored.

We do not yet have a clear understanding of how incidental visceral and emotional states may interact and exert influence over moral judgments in the moment, as the relationships between these variables are complex and multidirectional. Food deprivation can affect physiological arousal (e.g., Korbonits et al., 2004; Chan et al., 2007) and emotional processes (MacCormack and Lindquist, 2016), which are known to influence moral judgments regarding the harm of others (Damasio et al., 1990; Greene et al., 2001; Cushman et al., 2012; Parton and McGinley, 2019). Hunger also influences interoceptive processes and may even heighten our awareness of changes in cardiac arousal (Herbert et al., 2012). A heightened awareness of internal sensations associated with hunger/thirst may increase the availability of bodily cues (Domschke et al., 2010). Hunger states could therefore influence moral decision-making, e.g., by reducing the harshness of moral acceptability judgments (e.g., Vicario et al., 2018), but the direction of this effect has not been previously investigated with harm-based moral judgments. Emotional state is fundamentally linked with interoceptive processes and hunger (Macht and Simons, 2000; Barrett, 2016; MacCormack and Lindquist, 2016), and can affect moral judgments (e.g., Valdesolo and Desteno, 2006; Zhang et al., 2017b). People’s current emotional experiences could therefore modulate the relationship between hunger/thirst and moral decision-making. We also explored the influence of sex, age, and individual differences in anxiety for predicting moral judgments. Women and older people are more likely to reject harmful action in hypothetical moral dilemmas (Armstrong et al., 2019; McNair et al., 2019). Anxiety is associated with heightened cardiac arousal, which can affect how we process threatening information (Garfinkel and Critchley, 2016), and is a psychological correlate of both hunger (Herman et al., 1987) and IS (Domschke et al., 2010). The role of anxiety in moral decision-making appears mixed. Anxiety has shown to increase unethical behavior in some circumstances (Kouchaki and Desai, 2015), with trait anxiety and self-focused emotional distress demonstrating varying influences on moral judgments (Sarlo et al., 2014).

The current study aimed to assess the interdependent relationships between IS, hunger, and moral judgments of harm with the following research questions (the protocol was registered on the Open Science Framework; Brown et al., 2019).

R1. Does felt hunger or thirst bias responses to a moral judgment task?

R2. Does IS moderate the relationship between hunger/thirst and moral judgments of harm?

R3. Does emotional state moderate the relationship between hunger and moral judgments of harm?

R4. Does sex, age, and/or anxiety predict moral judgments of harm?

## Materials and Methods

### Design

This was a within-subjects cross-sectional study (*n* = 154) testing preregistered research questions and exploratory hypotheses in a series of regression analyses. Moral appropriateness and moral acceptability judgments were the dependent variables. Hunger, thirst, IS, incidental emotional state, and performance on the CRT (Frederick, 2005) were the independent variables. The influence of age, sex, and anxiety for predicting moral judgments was also explored.

### Measures

#### Demographics

Participants completed a brief demographic form indicating their sex, age, nationality, and ethnicity. Collecting sex data was preferred over gender, as physiological sex differences were more relevant because of known sex differences in interoceptive abilities. Experience in mindfulness/mediation practice was collected as a control variable because of its associations with body awareness (Bornemann et al., 2015), which could inform interpretation of the results. The item read: “Are you an experienced meditator or regularly practice mindfulness?” with the following response options: No/Practice mindfulness or mediate occasionally/Yes, coded for analysis.

#### Health Questionnaire

A brief health questionnaire was used to assess participants’ general health on the day prior to and day of the experiment in the interest of managing any outliers that could influence the dependent and independent variables, e.g., feelings of nausea, sickness. Only one of the questions regarding “current state of health” was coded for analysis as it was deemed more relevant to the participant’s current emotional state. The item read: “How is your overall health at this moment?” and response options included: Very bad/Unwell, Slightly unwell, No complaints, Fine, Very good. These were numerically coded 1 to 5 before the analysis to create a measure of “current health.”

#### Anxiety

State and trait anxiety were measured using the State and Trait Anxiety Scale (Spielberger and Gorsuch, 1983). This consists of two identical 20-item scales that ask participants to rate how they feel *right now* (state anxiety) and how they feel *in general* (trait anxiety). Participants were asked to indicate their agreement (Not at all/Somewhat/Moderately so/Very much so), with 20 different statements, e.g., “I feel calm,” “I feel tense,” “I feel at ease.” The scales include positively and negatively coded items to calculate two cumulative scores representing state and trait anxiety.

#### Interoceptive Sensibility

Interoceptive sensibility (IS) concerns individuals’ beliefs about their sensitivity to normal bodily processes (Garfinkel and Critchley, 2013; Ferentzi et al., 2018) and was measured using the Private Body Consciousness subscale of the Body Consciousness Questionnaire (Miller et al., 1981). This subscale offers a parsimonious measure of IS, focusing specifically on bodily sensations, and is commonly used in interoception research e.g., (Werner et al., 2009; Sze et al., 2010; Ainley and Tsakiris, 2013). The entire Body Consciousness Questionnaire (Miller et al., 1981) was used in the interest of maintaining scale validity. Only scores for the Private Body Consciousness subscale (PBCQ) were calculated for analysis, which includes five questions measuring how often people typically notice or pay attention to interoceptive sensations. Subscale items include the following: “I know immediately when my mouth or throat gets dry,” “I am sensitive to internal bodily tensions,” and “I am quick to sense the hunger contractions in my stomach.” Participants indicated how characteristic each statement was of themselves on a scale (extremely uncharacteristic/uncharacteristic/neutral/characteristic/extremely characteristic). Items were numerically coded 1 to 5, resulting in a maximum possible score of 25. Mean scores were calculated for all participants before analysis.

#### State Emotion

State emotion was measured using the Positive and Negative Affect Scale (PANAS; Watson et al., 1988). Positive affect (PA) and negative affect (NA) represent two independent subscales of subjective emotional experience. Each subscale consists of 10 items and demonstrates high internal reliability (PA: Cronbach α = 0.89, NA: Cronbach α = 0.85; Crawford and Henry, 2004).

#### Moral Judgment Stimuli

Conway and Gawronski’s (2013) moral dilemma stimuli were used to assess independent response-tendencies of harm aversion and outcome maximization separately. Harm aversion represents the tendency to reject the moral appropriateness of carrying out harmful actions regardless of the consequences of harm, whereas outcome maximization represents the tendency to condone the moral appropriateness of harmful action, if harm results in a greater good overall (e.g., saving more lives). Participants respond with a yes or no response (Table 1). Their text-based procedure includes 20 moral dilemmas made up of 10 story pairs, which are matched in story content, but the amount of “greater good” resulting from a harmful action is manipulated. This makes it possible to measure harm-aversion tendencies while controlling for people’s tendency to maximize outcomes. A harm-aversion and outcome-maximization score is calculated for each person, based on the probability that they accepted/rejected harm when harm resulted in a “greater good” and when it did not (for full probability equations, see Conway and Gawronski, 2013).

**TABLE 1:** Matched pair of moral dilemma stimuli from Conway and Gawronski’s (2013) moral dilemma task.

Causing harm maximizes outcomes	Causing harm does *not* maximize outcomes
You are a soldier guarding a border checkpoint between your nation and one troubled by insurgent violence. You notice a young man in a cheap car approaching the checkpoint with a determined look on his face. You suspect he means to bomb the checkpoint, **killing all the soldiers inside.** He is rapidly approaching your station. **Question:** Is it appropriate for you to shoot and kill the approaching man? Yes/No	You are a soldier guarding a border checkpoint between your nation and one troubled by insurgent violence. You notice a young man in a cheap car approaching the checkpoint with a determined look on his face. You suspect he means to cross the border in order to **work illegally inside your country**. He is rapidly approaching your station. **Question:** Is it appropriate for you to shoot and kill the approaching man? Yes/No

#### Moral Acceptability Ratings

Moral acceptability ratings of allocentric ethical violations (Schnall et al., 2008) or moral goodness ratings of harmful actions (Sarlo et al., 2014) provide a useful scale measure of the strength of people’s judgments of harmful actions. Here we implemented a moral acceptability measure, to capture the strength of people’s allocentric moral judgments when harmful action results in a greater good and when it does not. Following each of the moral dilemmas, we asked participants to judge the moral acceptability of the harmful actions proposed in the previous moral dilemma (Table 1). The item read: “How morally acceptable or morally unacceptable do you find the proposed action to be?” Response options included: 1 = completely unacceptable, 2 = moderately unacceptable, 3 = slightly unacceptable, 4 = neither acceptable nor unacceptable, 5 = slightly acceptable, 6 = moderately acceptable, 7 = completely acceptable (adapted from Schnall et al., 2008).

#### Hunger and Thirst

Two separate, single-item visual analog scales were used to assess self-reported sensations of hunger and thirst on a scale of 1 to 9: “How hungry/thirsty do you feel at this moment?” (1 = not at all, 9 = extremely hungry/thirsty). Hunger and thirst were assessed last to avoid any priming effects before the moral judgment task.

#### Cognitive Reflection Task

The original CRT (Frederick, 2005) assesses participant’s ability to override intuitive or “gut” responses to counterintuitive problems. The task involves three questions that have both an intuitive and correct answer, e.g., “A bat and a ball cost $1.10 in total. The bat costs $1.00 more than the ball. How much does the ball cost?” Participants manually typed their answers, and response time was not capped. Successful performance on this task requires further deliberation of the questions to determine the correct solutions, and therefore, better performance is associated with a greater ability to override the “intuitive” or more obvious answer. This measure aimed to capture participant’s intuitive versus analytic decision-making tendencies when faced with counterintuitive problems. There are many possible scoring methods for the CRT. As such, both the “regular” scoring method (totaling only correct answers) and the “intuitive” scoring method (totaling only intuitive answers and disregarding incorrect answers) were used (Erceg and Bubić, 2017), to inspect correlations between these alternative calculations.

### Procedure

Following approval from University of Bath Psychology Ethics Committee, 154 participants were recruited online via advertisements displayed on University of Bath research participation portal and social networking sites. The experiment was developed in Qualtrics and accessible via an anonymous web link. All partially completed questionnaires were excluded from analysis. We exceeded our target sample size of 120 participants, which was based on *a priori* power calculations using G^∗^Power for multiple linear regression models, assuming α = 0.95, β = 0.8, and *f*^2^ = 0.10 (df = 8). Inclusion criteria for participation were guided by a literature review of physiological and psychological confounds known to influence the primary independent variables, namely, hunger, thirst, and interoception. Participants were required to be aged 18 + years; with no current mental health issues; no history of disordered eating, diabetes, thyroid conditions, gastrointestinal or heart conditions, or previous surgery to those areas; and no current health conditions or medication that affected diet, weight, or exercise. Eligibility criteria were emphasized on the research advertisements and participant information sheet.

Potential participants accessing the experiment in Qualtrics were first presented with a study information sheet. They were then asked to confirm they met eligibility requirements and encouraged to contact the experimenter with any questions or concerns about taking part. Participants then completed an online consent form and were made aware they could enter their names into a prize draw at the end of the experiment in exchange for their participation. Participants worked through a series of questionnaires in the order outlined below with instructions provided before each questionnaire. The experiment took roughly 30 min to complete and could be mostly carried out at the pace of the respondent. The moral dilemma task was the only timed element of the experiment, whereby the text for each moral dilemma story would time out after 45 s and was followed by the moral judgment questions. Participants could advance to the questions after 20 s with a button click. This ensured reading time for each moral dilemma was roughly standardized and was clearly signposted in the instructions before starting the task. Upon completion of the study, participants were thanked for their time and provided with some further information about the study and experimenter contact details. They were then asked if they would like to enter the prize draw to win one of four 25 Amazon vouchers, by entering their details via an anonymous link to a raffle survey in Qualtrics.

## Results

### Data Reduction and Descriptive Analysis

The sample was 31.8% male, and the ages of participants ranged from 18 to 70 years [median = 31, standard deviation (SD) = 12.21]. Statistical analysis was carried out with SPSS v.24. A Pearson bivariate correlation analysis including all of the variables was conducted first, followed by a series of ordinary least squares regression analyses to address preregistered and exploratory hypotheses. The SPSS scripts for moderation, mediation, and conditional process analyses (PROCESS) were adopted from Hayes (2018). For all moderation analyses carried out in PROCESS, interactions are probed at the 16th, 50th, and 84th percentiles by default. As females were considerably overrepresented in this sample, a bootstrapping method was adopted for all regression analyses (5,000 × bootstrapping samples, 95% confidence interval) to generate standard error estimates that do not rely on parametric assumptions (Hayes, 2018).

#### Dependent Variables: Moral Judgments and Moral Acceptability Ratings

The four moral judgment–dependent variables included (1) harm aversion and (2) outcome maximization tendencies and moral acceptability ratings for (3) congruent and (4) incongruent trials. Raw harm-aversion and outcome-maximization scores were standardized into *Z* scores as suggested (see Supplementary Material; Conway and Gawronski, 2013). As expected, harm-aversion and outcome-maximization scores showed only weak negative correlation (*r* = −0.092, *p* = 0.259), confirming the independence of these response tendencies. To explore whether people judged harmful actions (from an allocentric perspective) as more morally acceptable for trials where harm maximized outcomes and when it did not, moral acceptability ratings for the harmful actions proposed in each moral dilemma were averaged for trials where harm did not maximize outcomes (congruent) and trials where harm maximized outcomes (incongruent) (Table 1). This resulted in two average moral acceptability scores for each participant: (1) acceptability_incongruent and (2) acceptability_congruent. Each acceptability score represented 10 moral acceptability ratings. Moral acceptability scores for congruent and incongruent trials were strongly positively correlated (*r* = 0.640, *p* < 0.001). This indicates people were relatively consistent in how morally acceptable they judged harmful actions to be from an allocentric perspective across all trials, when harm maximized outcomes and when it did not.

The distribution of studentized residuals of the dependent variables was inspected. Outcome-maximization scores and acceptability_incongruent scores were fairly normally distributed. Harm-aversion scores sat slightly higher than the mean on average; however, only mild skewness was identified. A log10 transformation was carried out on acceptability_congruent scores to adjust for a strong positive skew. For all regression analyses, a casewise diagnostic was performed on studentized residuals to identify outliers affecting the values of the estimated regression coefficients. Only three outliers ± 3 SDs were identified overall and removed from the associated regression analysis. A Cook distance and Levene test confirmed no leverage values or unusual data points in each regression model. All other regression assumptions were met.

#### Scale Reliability

The state emotion and trait and state anxiety measures showed high internal reliability. Coefficient *a* values were 0.87 for positive affect 0.89 for negative affect (0.82 for the entire PANAS measure) 0.94 for state anxiety, and 0.95 for trait anxiety. Adequate internal consistency was found for the private body consciousness subscale (five items) of the Body Consciousness Questionnaire (*a* = 0.65) and is comparable to prior research (Christensen et al., 1996). Scores for the CRT were coded for the presence of correct answers (regular scoring) and intuitive answers (intuitive scoring) and demonstrated very high negative correlation (*r* = −0.910, *p* < 0.001). Correlations for the independent variables can be found below in Table 2.

**TABLE 2:** Pearson coefficients for all independent variables in vertical order: hunger, thirst, state anxiety, trait anxiety, positive affect, negative affect, interoceptive sensibility, Cognitive Reflection Task score (regular), age, sex (male = 1, female = 2), current health (*p < 0.05; **p < 0.01).

	Thirst	State anxiety	Trait anxiety	Positive affect	Negative affect	Interoceptive sensibility	CRT	Age	Sex	Health
Hunger	0.289**	–0.30	–0.089	–0.031	–0.071	0.152	–0.069	0.000	0.041	0.050
Thirst		0.165	0.136	–0.117	0.140	0.247**	0.012	–0.061	0.064	–0.154
State anxiety			0.726**	−0.197*	0.454**	0.038	–0.045	−0.318**	0.119	−0.430**
Trait anxiety				−0.284**	0.454**	0.121	–0.008	−0.294**	0.227**	−0.325**
Positive affect					–0.072	–0.125	0.057	0.143	–0.156	0.204*
Negative affect						0.083	–0.023	−0.205*	0.230**	–0.148
Interoceptive sensibility							−0.216**	–0.004	0.093	–0.072
CRT								0.047	–0.121	–0.029
Age									−0.253**	0.155
Sex										–0.040

#### Hunger and Thirst

The mean hunger rating was 3.24 (SD = 2.09) and 4.07 for thirst (SD = 1.96), and the mode being 1 for hunger and 3 for thirst. As expected, hunger and thirst were positively correlated (*r* = 0.294), and both scores positively predicted how many hours it had been since participants reported eating. Hunger and thirst scores were relatively normally distributed, although participants typically reported less felt hunger than the median response option. Thirst was positively correlated with interoception (*r* = 0.247, *p* < 0.001), suggesting that people who were more likely to focus on internal sensations were also more likely to report subjective experiences of thirst.

#### Anxiety, Emotion, and Interoceptive Sensibility

Both state and trait anxiety strongly positively correlated with negative affect and were negatively correlated with positive affect (Table 2), which is expected as subjective arousal comprises a core component of affective experience (Russell and Barrett, 1999). More anxious people were more likely to report feeling unwell, and although the direction of the relationship is unclear, correlation between anxiety and health-related concerns is consistent with other work in this field (Domschke et al., 2010; Paulus and Stein, 2010). A noteworthy observation was that self-reported frequency of mindfulness practice (see *Demographics*) was positively correlated with IS (*r* = 0.274, *p* = 0.001), suggesting people with a tendency to focus on bodily sensations engaged in mindfulness more often. Therefore, people exhibiting a greater tendency to notice bodily sensations in this study may have demonstrated a healthier, more adaptive attentional style toward bodily sensations as opposed to a more anxious preoccupation with bodily sensations.

### Analyses

#### R1. Hunger, Thirst, and Moral Judgments of Harm

R1 tested whether felt hunger or thirst biased moral judgments of harm. Hunger and thirst ratings were entered as predictor variables in four multiple linear regression models. Outcome maximization, harm aversion, and moral acceptability for congruent (acceptability_congruent) and incongruent trials (acceptability_incongruent) were the dependent variables. Contrary to our hypothesis, neither hunger (*b* = 0.072, *p* = 0.402) nor thirst (*b* = 0.036, *p* = 0.675) predicted participant’s harm aversion scores (*R*^2^ chng = 0.010, *F*(2,149) = 0.597, *p* = 0.552). Moreover, hunger (*b* = 0.043) and thirst (*b* = −0.043) did not predict participant outcome-maximization scores (*R*^2^ chng = 0.003, *F*(2,149) = 0.195, *p* = 0.823). Therefore, participants who were more hungry or thirsty were not more or less likely to accept/reject harm or maximize outcomes on the moral dilemma task than those who were less hungry or thirsty. Hunger (*b* = 0.118, *p* = 0.170) and thirst (*b* = −0.052, *p* = 0.544) also did not predict acceptability_incongruent scores (where harm results in a “greater good”) (*R*^2^ chng = 0.013, *F*(2,148) = 0.973, *p* = 0.380). Hunger did negatively predict acceptability _congruent scores (*b* = 0.226, *p* = 0.008) in the model (*R*^2^ chng = 0.049, *F*(2,148) = 3.841, *p* = 0.024) but thirst did not (*b* = −0.116, *p* = 0.167). Therefore, hungrier participants were more likely to judge the moral acceptability of harmful actions as less “wrong,” but only for trials where harmful actions resulted in no greater good overall. Finally, as hunger and thirst are related sensations often physiologically interlinked, a *post hoc* mediation analysis was conducted to assess whether hunger (*a*) influenced acceptability_congruent scores through experiences of thirst (*b*). A bootstrap confidence interval for the indirect effect (*ab* = −0.0421) included zero (-0.0059 to 0.0005), indicating that hunger did not influence moral acceptability ratings on congruent trials through related experiences of thirst. Despite the positive correlation between hunger and thirst ratings, how thirsty participants felt did not appear influential for moral acceptability ratings across all trials.

#### R2. Moderating Role of Affective State

In R2, we explored the moderating role of affective state in the relationship between hunger and moral judgments of harm. Against our predictions, no significant correlations were found between hunger, thirst, and positive or negative emotional state or between emotional state and moral judgments (Table 2). Non-significant relationships between hunger/thirst and moral judgments were not probed for moderation effects of emotional state. A moderating role of positive affect (*R*^2^ chng = 0.0118, *F*(1,147) = 1.82, *p* = 0.1793) and negative affect (*R*^2^ chng = 0.012, *F*(1,147) = 1.853, *p* = 0.1755) was not found in the relationship found between hunger and acceptability_congruent ratings found in R1. A further mediation analysis was carried out to rule out the possibility of hunger influencing acceptability_congruent ratings through changes in emotional state. Bootstrap confidence intervals of the indirect effect of hunger through positive affect (-0.0012 to 0.0012) and negative affect (-0.0013 to 0.0008) on acceptability_congruent ratings were entirely below zero, ruling out any mediation effects. Together this indicates the influence of hunger on moral acceptability ratings of unprofitable harmful acts cannot be explained by hunger-associated changes in emotional state. A two-step hierarchical regression controlling for the effects of positive affect, negative affect, and state anxiety also confirmed hunger significantly influenced acceptability_congruent ratings (*b* = 0.197, *p* = 0.016). Therefore, the influence of hunger on non-profitable judgments of harm was independent of affective experience.

#### R3. Moderating Role of Interoceptive Sensibility

In R3, we proposed that greater IS (tendency to focus on bodily sensations) could increase the availability of visceral sensations associated with hunger or thirst that could moderate the relationship between hunger/thirst and moral judgments. Contrary to R3, a moderation analysis yielded no interaction effect between hunger and interoception for outcome-maximization tendencies (*R*^2^ chng < 0.001, *F*(1,147) = 0.054, *p* = 0.817), harm-aversion tendencies (*R*^2^ chng = 0.0179, *F*(1,147) = 2.719, *p* = 0.101), acceptability_incongruent scores (*R*^2^ chng = 0.0064, *F*(1,147) = 0.964, *p* = 0.328), or acceptability_congruent scores (*R*^2^ chng = 0.003, *F*(1,147) = 0.455, *p* = 0.501). Similarly, no moderation effect of interoception was found between thirst and outcome maximization (*R*^2^ chng = 0.000, *F*(1,147) = 0.064, *p* = 0.799), harm-aversion tendencies (*R*^2^ chng = 0.0026, *F*(1,147) = 0.395, *p* = 0.531), acceptability_incongruent scores (*R*^2^ chng < 0.001, *F*(1,147) = 0.002, *p* = 0.961), or acceptability_congruent scores (*R*^2^ chng < 0.001, *F*(1,147) = 0.005, *p* = 0.979). Therefore, the influence of sensations of hunger or thirst on participant’s moral acceptability and moral appropriateness judgments did not vary as a function of their tendency to focus on bodily sensations.

#### R4. Influence of Sex, Age, and Anxiety on Moral Judgments

Age and sex were inputted as predictors in multiple linear regression models of all of the dependent variables (harm aversion, outcome-maximization tendencies, acceptability_congruent, and acceptability_incongruent ratings). We found participants’ age (*b* = 0.216, *p* = 0.009) and sex (*b* = 0.220, *p* = 0.008) significantly predicted harm-aversion tendencies, with females and older participants showing greater harm-aversion tendencies irrespective of the consequences of harm (*R*^2^ = 0.071, *F*(2,148) = 5.656, *p* = 0.004). Neither age nor sex predicted outcome-maximization tendencies, i.e., acceptance of harm in the interests of the “greater good” (*R*^2^ = 0.003, *F*(2,148) = 0.215, *p* = 0.807). Age and sex also did not predict acceptability_congruent (*R*^2^ = 0.022, *F*(2,148) = 1.626, *p* = 0.200) or acceptability_incongruent ratings (*R*^2^ = 0.026, *F*(2,148) = 1.959, *p* = 0.145). Therefore, age and sex did not influence how morally acceptable people judged harmful actions to be, despite the differences in harm-aversion tendencies overall. In partial support of the role of anxiety in moral judgments, state anxiety negatively correlated with harm aversion (*r* = −0.177, *p* = 0.03), indicating people who were more anxious at the time of the experiment were less likely to reject causing harm in the moral dilemmas. However, a hierarchical regression model confirmed that state anxiety did not significantly predict harm-aversion scores when controlling for the effects of sex and age (*R*^2^ chng = 0.091, *F*(3,147) = 4.911, *b* = −0.150, *p* = 0.078). State anxiety was significantly negatively correlated with age (*r* = 0.318, *p* < 0.01), with younger people more likely to report both state and trait anxiety. Therefore, age appears to account for much of the variation in state anxiety that predicted harm rejection judgments toward the moral dilemmas.

### Exploratory Analyses

#### EH1. CRT Performance and Moral Judgments

We tested the hypothesis that more accurate performance on the CRT task would positively predict more utilitarian response tendencies in line with prior research (Baron et al., 2015; Byrd and Conway, 2019).

Contrary to EH1, CRT performance showed significant positive correlation with harm aversion (*r* = 0.235, *p* = 0.004) but not outcome-maximization tendencies (*r* = 0.048, *p* = 0.562). As gender differences have been found for CRT performance (Ring et al., 2016), a multiple linear regression controlling for the effects of age and sex confirmed that CRT scores significantly predicted harm-aversion tendencies (*R*^2^ chng = 0.135, *F*(3,147) = 7.655, *b* = 0.255, *p* = 0.001). This finding was sustained when inputting alternative CRT scores representing the presence of “intuitive” answers as opposed to correct answers. Therefore, participants who were more likely to provide “intuitive” answers on the CRT were more likely to accept causing harm in moral dilemmas, irrespective of the outcomes.

#### EH2. CRT Performance, Interoceptive Sensibility, and Harm Aversion

Following EH1, we explored the predictive relationship between IS and the performance on the CRT task. We further investigated the possibility of a mediation effect of IS through intuitive decision-making processes (captured by the CRT) on harm-aversion responses.

Following identification of moderate correlation between CRT scores and IS (*r* = −0.216, *p* = 0.008), a linear regression model confirmed that higher IS predicted more incorrect and intuitive responses on the CRT (*R*^2^ chng = 0.046, *F*(1,149) = 7.264, *p* = 0.008). A mediation analysis explored the presence of an indirect effect of IS (*a*) on harm-aversion scores, through more “intuitive” decision-making processes on the CRT (*b*). The direct effect of interoception on harm-aversion scores was not significant (*t* = –0.272, *p* = 0.786). However, a bootstrap confidence interval of the indirect effect (*ab* = −0.0802) was entirely below zero (−0.1718 to −0.0110), suggesting that people with a greater tendency to focus on internal sensations provided more intuitive responses on the CRT and were more likely to condone harmful actions. Therefore, IS explains a significant amount of variance in “intuitive” CRT responses, which subsequently predicts participants acceptance of harmful actions.

## Discussion

An unexpected and novel discovery in this study was that hunger bias appeared uniquely influential for acceptability judgments of non-profitable harmful actions, whereas “intuitive” decision-making tendencies exclusively predicted appropriateness judgments of harm. These independent effects suggest that a metaphorical “gut instinct” and gut-related visceral experiences of hunger have distinct influences on harm-based moral cognition. We do have the capacity to be morally hypocritical; although we may judge an action to be morally appropriate, we can equally judge that act to be morally unacceptable (Tassy et al., 2013). Framing questions such as “is it appropriate to…?” versus “how morally acceptable do you find…?” assume different perspectives of the judge, and inconsistencies have been found between these types of judgments previously (Tassy et al., 2013; Pletti et al., 2017). Choice judgments such as “Would you do… in order to…?” involves forming a judgment from an egocentric perspective and makes self-relevant consequences more salient (Sood and Forehand, 2005; Tassy et al., 2013). Choice judgments are akin to the moral appropriateness judgments in this study, which encouraged people to adopt the perspective of the person carrying out the harmful action in the story (Table 1), whereas moral acceptability judgments provide a more abstract or allocentric perspective to evaluate a harmful act and create distance from the self and refer to the moral acceptability judgments in this study (Frith and De Vignemont, 2005). These two types of judgments may rely on distinct neural bases associated with differing degrees of agency. For example, egocentric moral judgments, but not allocentric, have been associated with activation of the amygdala, suggesting these judgments activate emotional processes associated with weighing up the consequences of our own actions for ourselves (Berthoz et al., 2006). Moreover, experiencing oneself as the cause of action has shown to activate areas of the anterior insula (Adolfi et al., 2017), whereas experiencing someone else as the cause of action is associated with activation of the inferior parietal cortex (Farrer and Frith, 2002). The importance of “where we are” in relation to harm has also come to light in virtual-reality studies that find discrepancies between hypothetical moral judgments people make and the harmful behaviors they perform when confronted with more realistic moral dilemmas (e.g., Francis et al., 2016).

In R1, we found less hungry people rated harm as more wrong in instances where harm did not result in any “greater good” overall, although this predictive relationship was relatively weak. This suggests hunger may be uniquely influential for allocentric judgments about unprofitable harmful acts. In line with prior research, the physiological changes associated with hunger states could bias how severely we judge the acceptability of moral violations from an allocentric perspective (Vicario et al., 2018), but the exclusivity of this effect for non-profitable harmful actions is a novel finding for harm-based moral dilemmas. Arguably, acceptability judgments for the trials where harm did not result in a “greater good” provides a judgment of the “wrongness” of excessive harm, as there is no moral justification to judge harm that is without benefit as morally acceptable. However, clearly people did judge certain types of harm to be more acceptable than other types, and this appears to have been influenced by their level of hunger. This is discussed further below. Moral appropriateness judgments, however, may reflect more stable aversions people have to characteristically harmful actions (action aversion) and witnessing the pain of others (outcome aversion) (Miller et al., 2014), which are more impervious to temporary hunger states. Alternatively, the binary yes/no option may simply prevent us from understanding the true strength of appropriateness judgments. Surprisingly, in R3, we found emotional state did not moderate the relationship between hunger and moral judgments. IS also did not moderate any relationships between hunger, thirst, and moral judgments, contrary (R2). Null findings for R2 and R3 suggest that hunger “acted alone” to influence non-profitable moral acceptability judgments of harm and cannot be explained by differences in people’s tendency to focus on visceral sensations such as hunger or incidental emotional state (e.g., Valdesolo and Desteno, 2006).

Perhaps most surprising was that CRT performance (directly) and IS (indirectly) predicted people’s harm-aversion tendencies. This may suggest a discrete influence of intuitive decision-making processes and IS for judgments of harm when adopting an egocentric viewpoint of the actor causing harm. This finding contradicts exploratory hypothesis EH1 and prior research showing a positive relationship between CRT performance and outcome maximization or “utilitarian” tendencies (Baron et al., 2015; Byrd and Conway, 2019). Although a logical reflection measure has correlated with harm-aversion tendencies before, arithmetic reflection (assessed by the CRT) has not (Byrd and Conway, 2019). The fact that more arithmetically correct answers on the CRT predicted the rejection of harmful action when harm resulted in a “greater good” and when it did not challenges the view that a more arithmetic focus is responsible for moral judgments that prioritize the number of lives saved (Byrd and Conway, 2019; Patil et al., 2020). An association between CRT performance and harm aversion is also counterintuitive to dual-process perspectives (Greene et al., 2001) that propose the rejection of harmful actions is associated with a faster, more emotional decision-making pathway that we might expect to negatively correlate with intuitive responses on the CRT (e.g., Kahneman and Frederick, 2002). People with higher IS were more likely to provide “intuitive” answers on the CRT task, suggesting greater bodily awareness impeded successful performance on this task. Furthermore, in EH2, we found IS indirectly predicted harm-aversion tendencies through its influence on CRT performance, whereby heightened IS appeared to reduce people’s ability to resolve counterintuitive problems on the CRT, which subsequently increased the likelihood they would condone harmful actions on the moral dilemma task. There is some support for the notion that an awareness of somatic states could actually enhance our representations of ourselves in relation to our moral responsibilities (Immordino-Yang, 2011), but the findings here suggest a heightened focus on visceral sensations may somehow contribute to a weakening of our aversion to harmful actions.

In R4, we found age and sex were the strongest predictors of harm aversion but not outcome-maximization tendencies. As found previously, older participants and female participants were most likely to reject causing harm (Armstrong et al., 2019; McNair et al., 2019), but these age and sex differences did not extend to moral acceptability ratings – a distinction that has not been clarified before. There was partial support for a role of state anxiety in predicting harm-aversion tendencies, with more anxious people more likely to accept causing harm regardless of the outcomes. This is somewhat consistent with a previous finding (Kouchaki and Desai, 2015) and is potentially due to how anxiety influences how we process threatening information (see Garfinkel and Critchley, 2016). However, the predictive value of state anxiety appeared to be mostly explained by variation in age, with younger people significantly more likely to report higher levels of state anxiety.

### Hunger and Moral Acceptability Ratings

We did find hungrier people were more likely to judge non-profitable harmful actions as more morally acceptable, although the magnitude of effect was relatively weak and should be interpreted with caution. An absence of a relationship between hunger and state anxiety suggests these appraisals were not based on hunger-induced arousal (e.g., Korbonits et al., 2004; Chan et al., 2007). Indeed, hunger may not always induce physiological arousal in a negative sense (e.g., Michalsen, 2010), and hunger and state anxiety were, in fact, slightly negatively correlated in this study. Psychophysiological arousal has shown to predict an aversion to harmful actions (Cushman et al., 2012). Therefore, if arousal cues were reduced in hungrier individuals, it is possible this lessened the severity of their acceptability judgments and is consistent with the finding that hunger can actually reduce threat tolerance and promote riskier decision-making in animals (Ghosh et al., 2016). As the majority of people reported lower levels of hunger, it is possible that our sample did not include enough “very-hungry” participants to generate the hormonal and physiological responses associated with hunger-induced arousal. This subsequently reduces the probability of observing individual differences in state anxiety or negative affect associated with hunger that may have been influential.

As people who were less hungry reported to have eaten more recently, a “fullness”-based explanation is perhaps more likely and is consistent with some prior research (Vicario et al., 2018). Nausea symptoms often correlate with post-eating gastric emptying (Halawi et al., 2017) and can be interpreted emotionally as disgust (Tracy et al., 2019), which can influence moral judgments (see Haidt et al., 1994). However, this is a novel finding for harm-based moral judgments (Horberg et al., 2009). Nevertheless, this explanation is consistent with research finding positive correlations between hunger and acceptance of moral violations (Vicario et al., 2018), and between disgust sensitivity and disapproval of moral violations (Horberg et al., 2009; Vicario and Rafal, 2017). Unfortunately, as we did not measure disgust or fullness, these hypotheses remain speculative, although only 3% of the sample reported any nausea or gastrointestinal distress in a prestudy health questionnaire. Importantly, nausea associated with gastric dysrhythmias is not unique to visceral signaling processes associated with eating and can occur during hunger states and stomach emptiness (see Levine, 2005 for a review). However, why exactly hunger was influential only for moral acceptability judgments of harm that did not result in any “greater good” overall is unclear and requires further investigation.

### Interoceptive Sensibility

Interoceptive sensibility did not moderate the relationship between hunger and acceptability judgments of non-profitable harm (R2). As people’s tendency to focus on visceral sensations did not change the relationship between hunger and moral judgments, this implies that the psychophysiological processes proposed to underlie this relationship (e.g., Vicario et al., 2018) do not strengthen with higher levels of attention directed toward internal sensations. However, it is conceivable that people with higher levels of IS had lower thresholds for detecting sensations of hunger and thirst (Stevenson et al., 2015) and were more likely to overestimate “true” homeostatic states of hunger/thirst. This was evident for thirst at least, as a moderate correlation between thirst and IS (*r* = 0.247, *p* < 0.01) indicated people with a greater sensitivity to bodily sensations were more aware of thirst-type visceral sensations. Similarly, hunger was positively correlated with IS (*r* = 0.152) but was non-significant. It could be argued that the hunger and thirst ratings scales provided a measure of IS themselves, as they asked people to consciously assess and report subjective visceral states, which will of course depend on the availability of this information. Although problematic levels of multicollinearity between hunger/thirst and IS were not identified in the regression analyses, if changes in IS were met with corresponding changes in hunger/thirst ratings, this would reduce the likelihood of observing any moderation effects of IS in the relationship between hunger and moral judgments. Future work using a larger sample could generate more statistical power to uncover any small effect sizes of IS in the link between hunger and moral acceptability judgments not found here.

### Role of Emotional States and Anxiety

Interestingly, neither anxiety nor emotional state correlated with hunger or thirst providing no support for any association between these constructs found previously (see MacCormack and Lindquist, 2016). Agreeing on the archetypical symptoms and psychophysiological experiences of hunger and thirst is challenging, because of variations in eating contexts (Ribeiro et al., 2009) and the variation of visceral and emotional expressions of hunger and thirst people report (e.g., Michalsen, 2010). For example, a large proportion of people do not experience abdominal emptiness when hungry (Harris and Wardle, 1987), and some even report positive psychological experiences from food deprivation (Watkins and Serpell, 2016), which could partially explain why we did not find the anticipated relationships between hunger, thirst, and emotional states in this study. Alternatively, the null finding for R3 is perhaps due to the low variation in hunger and thirst ratings in this sample.

State anxiety negatively predicted harm-aversion tendencies on the moral judgment task but fell from significance when controlling for age and sex. Trait anxiety did not correlate with harm-aversion tendencies, which contradicts an earlier finding (Zhao et al., 2016) and is surprising considering that measures of dispositional threat reactivity have predicted people’s aversion to harmful actions (Cushman et al., 2012), with momentary anxiety inductions having less of an effect on moral judgments (Zhao et al., 2016). Anxiety can facilitate the processing of threatening information (Mathews, 1990) and increase anticipation of aversive outcomes (Paulus and Stein, 2010) and may explain the negative association found between state anxiety and harm-acceptance tendencies found here. More anxious people may have perceived the hypothetical recipients of harm to be more threatening or considered the option of not carrying out harm (i.e., doing nothing) to be the riskier option compared to less anxious people, but the link between anxiety, physiological arousal, and moral judgments is likely much more complex. Moreover, as emotion and anxiety were measured before participants completed the moral dilemmas, we can only speculate that any incidental feelings of anxiety or emotion were experienced as unrelated to the task as opposed to a reaction to the potential consequences of their choices on the task (see Baumeister et al., 2012).

### CRT, Interoceptive Sensibility, and Harm Aversion

While interoceptive accuracy (on a heartbeat detection task) has shown to influence CRT performance under certain conditions (Lugo et al., 2017), a relationship between IS and CRT performance is novel. Empirical work surrounding IS and cognition is limited. There is some evidence to suggest IS can influence risk-taking behavior (Salvato et al., 2019), but this does not appear related to impulsivity in decision-making (Herman et al., 2018). IS also indirectly predicted harm-aversion tendencies through its influence on CRT performance. Both IS (Paulus and Stein, 2010) and egocentric moral judgments are associated with forms of self-referential processing (Sood and Forehand, 2005), which is one speculative explanation of the indirect association between IS and harm aversion in this study. People scoring higher on IS may engage in self-referential processing to a greater extent, which possibly reduces their inclination to engage in computationally demanding decision processes when faced with counterintuitive problems like the CRT. Therefore, these people may be more likely to rely on intuitive heuristics to form their answer (Kahneman and Frederick, 2002), which may also have consequences for moral decision-making. Consistent with this hypothesis, perhaps the most parsimonious account for why the CRT was a predictor of harm-aversion bias here is because it taps into our general tendency toward being “cognitive misers” – preferring the processing option that requires least energy expenditure (Tversky and Kahneman, 1974; Toplak et al., 2011). More intuitive responses on the CRT task could suggest reduced engagement or deliberation of items across the whole experiment, including the moral dilemmas where harmful actions do not result in a greater good overall. For these “congruent” dilemmas, weighing up the consequences of harmful action arguably requires slightly more scrutiny of the story content at times. If participants did not fully consider the specific content of the stories, they could have mistakenly condoned harmful actions due to misreading or overlooking story information, which would provide a negatively skewed measure of harm aversion for these people. Therefore, rather than poorer performance on the CRT task representing stable differences in intuitive thinking styles, it is possibly more a reflection of intuitive “preference” (Pennycook et al., 2016) based on the computational resource available or utilized at that moment (Toplak et al., 2011).

### Sex and Age Effects

In line with prior research using traditional moral dilemma paradigms, older participants demonstrated greater harm-aversion preferences which has been linked to a greater propensity to experience negative emotions (McNair et al., 2019) and/or a reduced ability to overcome affective cues when making judgments (Hess et al., 2000). Older participants in this study reported lower negative affect and state/trait anxiety than younger participants, and no age-related differences were found for IS. Therefore, incidental negative affect (unrelated to the task) or greater attentional focus toward affective cues in the body does not appear to underlie the finding here, but a more negative emotional response to the moral dilemma stimuli from older participants cannot be ruled out. Similarly, although some research has shown men demonstrate stronger utilitarian preferences (Tinghög et al., 2016), the finding that females scored similarly to men on utilitarian preferences but higher on harm-aversion tendencies is in line with cumulative research findings in this field (Armstrong et al., 2019). Explanations for gender differences typically center around differences in socialization practices (Wood and Eagly, 2012), as well as evolutionary pressures and physiological differences (see Armstrong et al., 2019 for a review), which may engender greater social and emotional responses to the prospect of harming others in women.

## Limitations

Online research into hunger and thirst has the advantage of gathering data from people in their natural eating environments but does not guarantee variation in visceral experiences or the presence of real physiological changes associated with hunger and thirst. It is possible that low variability of hunger (3.24 ± 2.09) and thirst ratings (4.07 ± 1.96) prevented us from uncovering individual differences in the impact of visceral and emotional states on moral decision-making. In addition, relying on self-report measures cannot provide an objective understanding of the physiological conditions accompanying these subjective states, and some research has found intraindividual inconsistencies using visual analog scales of appetite (e.g., Flint et al., 2000). One indication of reliability of our measure is that hunger significantly predicted hours since eating, providing the expected relationship between hunger states and reported ingestive behavior. Although we can never know what hungry or thirsty “feels” like to different people or guarantee a consistent impact of food-deprivation manipulations on visceral experiences (Michalsen, 2010; Stevenson et al., 2015), using fasting manipulations (Vicario et al., 2018) or measures of blood-glucose (Gailliot et al., 2007) would allow the objective investigation of the impact of homeostatic depletion on moral decision-making.

A second limitation was the measure of IS used PBCQ: Porges, 1993). Although popular in interoception research (e.g., Ainley and Tsakiris, 2013; Duschek et al., 2015; Garfinkel et al., 2015), the PBCQ provides a one-dimensional trait measure of perceptual awareness of bodily symptoms. An important distinction between body awareness attention styles (Mehling et al., 2018) is not captured by this measure and limits our understanding of IS in this context. A more negative attentional style is associated with anxiety and somatization (Domschke et al., 2010; Ginzburg et al., 2014), whereas a more adaptive attentional focus on the body can enhance self-regulatory processes associated with bodily sensations and is prevalent in mindfulness-style practices such as body scanning (Bornemann et al., 2015). As participants self-reported mindfulness practice positively correlated with IS, it is possible that participants on the higher end of the IS scale exhibited a “healthier” attentional focus on bodily sensations, which could explain the absence of any relationship between IS and anxiety. Future work using a Multidimensional Assessment of Interoceptive Awareness (Mehling et al., 2018) would provide a more nuanced understanding of the attentional and emotional regulation styles of people with higher levels of IS.

The CRT (Frederick, 2005) is a popular but controversial measure, inherently confounded with numeracy ability. It is possible the CRT provides an indication of people’s tendency to think in less effortful ways as opposed to reflecting stable individual differences in thinking styles (Toplak et al., 2011). A recent study found CRT scores did not reflect thinking styles or intuitive ability that was distinct from a general intelligence measure (Blacksmith et al., 2019), whereas other research suggests the CRT is valid for measuring reflective but not intuitive thinking styles (Pennycook et al., 2016). Ambiguity about whether the CRT taps into stable psychological constructs or more temporary psychological processes can make the interpretation of results difficult. Future replications could clarify whether the CRT’s power in predicting harm-aversion judgments was due stable individual differences in intuitive or rational thinking styles using measures such as the Rational–Experiential Inventory (Pacini and Epstein, 1999). Finally, our sample was a moderate size and well-represented in terms of age but contained a disproportionate number of women. Considering small effect sizes and several null findings in this study, a more substantial and representative sample would increase the power to uncover effects of visceral states and interoceptive processes on moral judgments if they do exist.

## Conclusion

When making difficult moral decisions, we may refer to a metaphorical “gut instinct” to explain our choices, a feeling we locate in our stomach area that steers us one way or another. Hunger is one such sensation fundamentally linked with our gastrointestinal system that appears to play a role in allocentric judgments of harmful acts and other moral transgressions, potentially due to its link with disgust (Schnall et al., 2008; Vicario et al., 2018). We also associate “gut feelings” with a felt sense of intuition. Intuition is easily linked with interoceptive processes, when we cannot consciously access the homeostatic valuations happening between the brain and body that can bias our decision-making processes (Damasio et al., 1996; Craig, 2015). Here, we found intuitive thinking preference on the CRT was associated with a tendency to pay attention to interoceptive sensations and a reduced aversion to harmful actions. Together, these findings suggest hunger bias and intuitive thinking preferences may represent independent processes shaping different types of moral judgments. It is possible that the presence of “intuitive” responses on the CRT may instead represent an absence of deliberative thinking processes (e.g., Toplak et al., 2011), and we speculated that increased monitoring of bodily sensations associated with body awareness could interfere with more effortful thinking processes due to the demand on attentional resources. Further work using validated measures of intuitive thinking (e.g., Pacini and Epstein, 1999) could clarify this supposition. Interestingly, incidental emotion and anxiety states did not moderate any relationship between hunger, interoception, CRT performance, and moral judgments. This suggests that emotional state at the time of making harm-based moral judgments did not provide any significant contribution to these effects, contrary to our hypotheses (Valdesolo and Desteno, 2006). Future work measuring people’s emotional state before and after the task could clarify whether a change in emotional state is more predictive of moral judgments than incidental emotional state. The findings of this study have gone some way in clarifying the influence of incidental visceral states, emotion, and IS on moral judgments of harm. Interoception is significantly understudied in morality research, which provides many more research opportunities to explore the complex relationships between interoceptive processes, emotion, and moral decision-making.

## Data Availability Statement

The raw data supporting the conclusions of this article will be made available by the authors, without undue reservation.

## Ethics Statement

The studies involving human participants were reviewed and approved by University of Bath Psychology Ethics Committee. The participants provided their written informed consent to participate in this study.

## Author Contributions

HB conceived the study idea, carried out the research, and conducted data analysis and manuscript writing. DS and MP supervised the entire project, piloted the research, and provided feedback and ideas on the study design, data analysis, and written manuscript. All authors contributed to the article and approved the submitted version.

## Conflict of Interest

The authors declare that the research was conducted in the absence of any commercial or financial relationships that could be construed as a potential conflict of interest.
